# Association of MRI-derived muscle-fat composition with knee osteoarthritis severity: a gender-stratified X-ray and MRI correlation study

**DOI:** 10.3389/fmed.2026.1816854

**Published:** 2026-06-23

**Authors:** Mohammad Al-Harbi, Anas Hamdoun, Zuhal Y. Hamd, Shaden Alhegail, Ammar Mallisho, Razan Salih, Mohamed Abuzaid, Wiam Elshami

**Affiliations:** 1 Internal Medicine Department, College of Medicine, Princess Nourah bint Abdulrahman University, Riyadh, Saudi Arabia; 2 Department of Medical Imaging, King Abdullah Bin Abdul-Aziz University Hospital, Riyadh, Saudi Arabia; 3 Department of Radiological Sciences, College of Health and Rehabilitation Sciences, Princess Nourah bint Abdulrahman University, Riyadh, Saudi Arabia; 4 Department of Medical Diagnostic Imaging, College of Health Sciences, University of Sharjah, Sharjah, United Arab Emirates; 5 Research Institute for Medical and Health Sciences, University of Sharjah, Sharjah, United Arab Emirates

**Keywords:** knee osteoarthritis, magnetic resonance imaging, radiography, Kellgren-Lawrence grading, muscle-to-fat ratio, body composition, cartilage degeneration, soft tissue changes

## Abstract

**Background:**

Knee osteoarthritis (OA) is a common musculoskeletal disorder characterized by the progressive degradation of articular cartilage, along with changes in surrounding soft tissues and bone structures. The knee joint is particularly susceptible to osteoarthritic changes, resulting in pain, functional impairment, and a significant decline in the quality of life for affected individuals. Plain radiography and MRI are considered the standards imaging modalities for diagnosis.

**Aim:**

This research aims to explore the correlation between soft tissue changes and the severity of knee OA, utilizing both traditional plain radiographs and advanced MRI techniques. The study also focuses on gender differences to provide insights into potential modifiable risk factors and therapeutic targets.

**Methods:**

A total of 232 patients underwent knee MRI and X-ray imaging for various clinical indications. Radiographs were classified according to the Kellgren-Lawrence scale, while MR images were used to assess body composition and degenerative changes in the knee.

**Results:**

Among the 232 participants, 54.7% were female and 45.3% were male, with most aged 30–44 years and categorized as obese (44.8%). The data revealed that muscle thickness remained consistent across BMI categories, with males generally exhibiting higher values than females. Fat thickness increased with higher BMI categories, with females consistently showing greater values than males. The muscle-to-fat ratio decreased as BMI increased, with males having higher ratio in the normal BMI category compared to females. Pearson’s correlation indicated that muscle to fat ratio has a weak negative correlation with soft tissue degenerative changes, and cartilage degenerative changes. Cohen’s Kappa coefficient analysis revealed fair agreement between binary classifications derived from X-ray and MRI findings. The McNemar’s test indicated a statistically significant difference in detection capabilities between the two techniques.

**Conclusion:**

The muscle-to-fat ratio and fat thickness significantly impact knee degenerative changes, particularly in females. This underscores the necessity for targeted interventions aimed at reducing fat thickness while preserving muscle thickness to enhance overall health outcomes. Although X-rays exhibit higher sensitivity, they have lower specificity compared to MRI; both diagnostic methods improve in accuracy as disease severity increases. The combination of MRI and X-ray further enhances the diagnostic accuracy for knee OA.

## 1 Introduction

Knee osteoarthritis (OA) is a prevalent musculoskeletal disorder characterized by the progressive degradation of articular cartilage, accompanied by changes in surrounding soft tissues and bone structures (1, 2). Among the various joints affected, the knee is particularly susceptible to osteoarthritic changes, leading to pain, functional impairment, and a significant decrease in the quality of life for affected individuals (3, 4).

Studies suggest that OA is a complex disorder influenced by a variety of pathogenic factors. While aging and obesity are well-established risk factors, disruptions in metabolic balance have also been associated with its onset. Inflammation plays a crucial role in the progression of OA, often originating from adipose tissue within the joint cavity (5).

One emerging area of interest in knee OA research is the role of soft tissue mass, including muscles, fat pads, and ligaments, in disease development and progression. Alterations in the composition and distribution of soft tissue mass have been implicated in the pathophysiology of knee OA, with both excess adiposity and muscle weakness identified as potential risk factors for disease onset and progression (6).

Traditionally, the assessment of knee OA has relied heavily on conventional imaging modalities such as plain radiography (X-ray), which primarily provide information about the bony structures and joint space narrowing characteristic of advanced OA. However, the limitations of plain radiographs in evaluating soft tissue structures have prompted the exploration of more advanced imaging techniques, notably magnetic resonance imaging (MRI). MRI offers superior soft tissue contrast, allowing detailed visualization and quantification of muscle volume, fat infiltration, and ligament integrity within the knee joint (7).

The integration of radiographic and MRI findings holds promise for elucidating the complex interplay between soft tissue characteristics and knee OA pathology. By correlating measurements of soft tissue mass obtained from both imaging modalities with clinical and functional outcomes. Furthermore, understanding the relationship between soft tissue alterations and disease progression may inform targeted therapeutic interventions aimed at preserving or restoring joint function in individuals with knee OA (8).

Despite the potential benefits of integrating radiographic findings and MRI assessments of knee OA in research, several challenges and unanswered questions remain. Methodological standardization, including the development of methods to measure and quantify soft tissue parameters, is essential to ensure the reproducibility and comparability of results across studies. Additionally, detailed investigations are needed to establish associations between changes in soft tissue characteristics and the onset of knee OA in different patient groups. This research aims to bridge this gap by exploring the correlation between soft tissue changes and the severity of knee OA, employing both traditional plain radiographs and advanced MRI techniques which could provide insights into potential modifiable risk factors or targets for therapeutic interventions (9).

## 2 Materials and methods

This retrospective observational study involves individuals with varying degrees of knee OA who underwent both knee MRI and X-ray between January 1 and December 31, 2024. The study was approved by the institutional review board of King Abdullah Bin Abdulaziz University Hospital (KAAUH).

The inclusion criteria were adult patients (age ≥ 16 years) who underwent both x ray and MRI imaging for suspected knee OA. Exclusion criteria were history of knee surgery, significant knee trauma, inflammatory joint diseases, or contraindications to MRI, as well as pediatric patients younger than 16 years old.

The X-ray imaging was performed using Philips Digital Diagnostic and Carestream DRX Evolution equipment. The X-ray projection of the knee joint were typically obtained by antero-posterior with 5 degrees cephalic angulation, and lateral with knee flection angle between 20 and 35 for a clear view of the joint gap between the patellofemoral and internal and external tibiofemoral joints (10, 11). The Kellgren-Lawrence grading guidelines, sometimes known as K-L grading, were used to assign grades to patients with knee OA (KOA) (12, 13).

**FIGURE 1:**
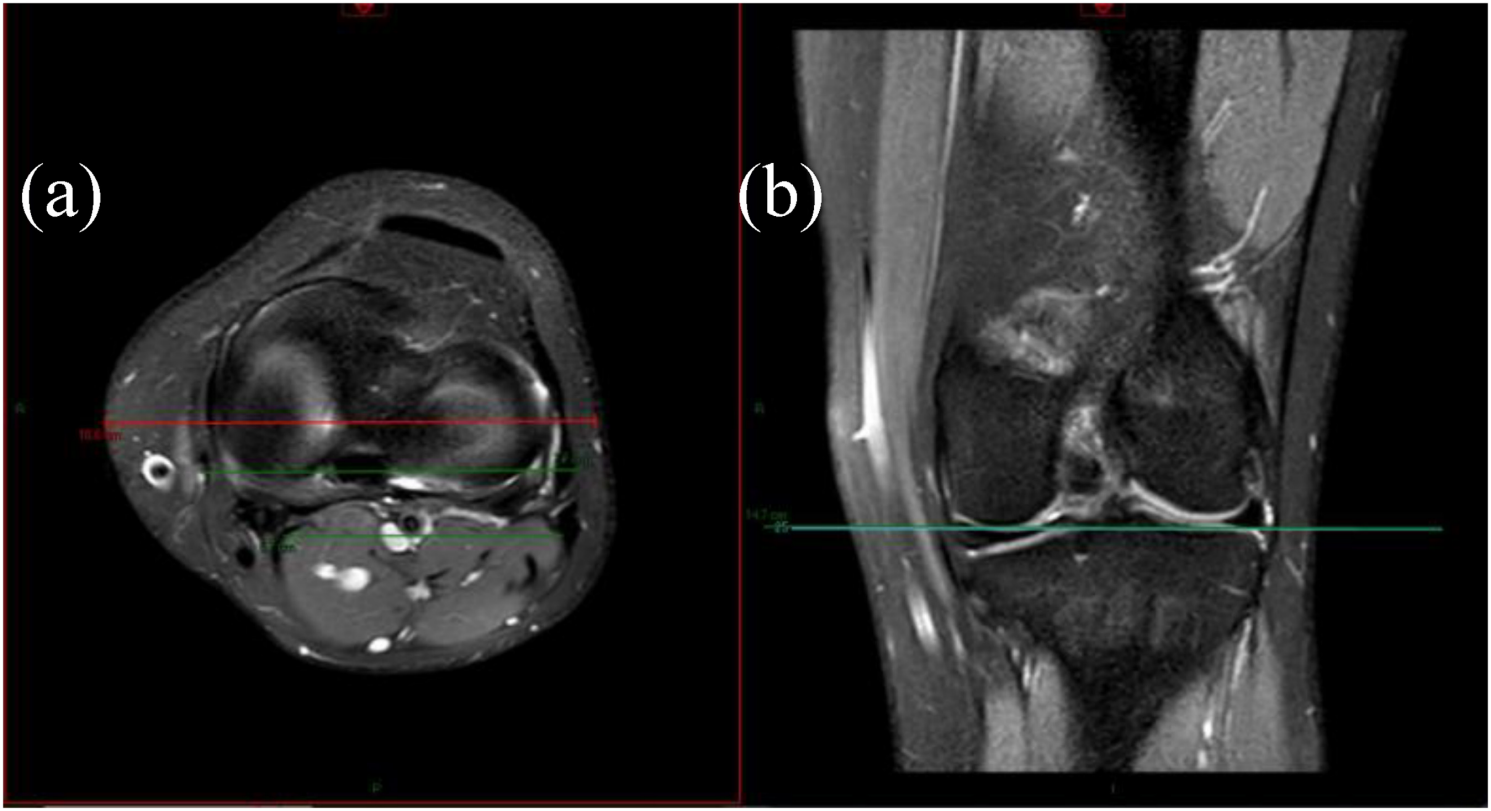
Knee MRI **(a)** axial and **(b)** coronal views showing the technique of measuring muscle and fat thickness. Total transverse width was measured from medial to lateral skin margins (red line). The osseous width of the femoral condyles was measured at the same level (green line), and the surrounding soft tissue thickness was estimated accordingly. Muscle thickness was measured at the posterior compartment (short green line).

MRI examination were performed using Siemens 3 Tesla MagnetomVida and Philips 1.5 Tesla Dstream Achieva machines were used. The protocol included sagittal, coronal, and axial PD-weighted fat-suppressed sequences, along with T1- and T2-weighted images. Typical parameters included slice thickness 3–4 mm, FOV 14–16 cm, and matrix 256–320. Multiplanar acquisition ensured comprehensive evaluation of joint structures. Figure 1 shows the periarticular soft tissue measurements on axial MRI at the level of maximal femoral condyle cross-sectional visualization demonstrating the measurement approach. Total limb width was defined as the distance between the medial and lateral skin margins (red line). Osseous width was measured as the cortical-to-cortical distance across the femoral condyles (long green line). Periarticular soft tissue thickness was indirectly calculated as the difference between total limb width and osseous width. Muscle thickness was measured in the posterior compartment as the maximal perpendicular distance across the muscle bulk (short green line). All measurements were obtained at a standardized anatomical level to ensure consistency and reproducibility across subjects. All measurements were performed using calibrated digital tools within the PACS system, with consistent image orientation and measurement direction maintained across all cases.

All measurements (X-rays and MRI scans) were independently reviewed by two radiologists with 11 and 15 years of experience[., followed by consensus. The radiologists were blinded to radiographic findings during MRI assessment. The average of the two measurements was used for analysis. Interobserver agreement was assessed separately for quantitative and categorical measures. For quantitative measurements (muscle and fat thickness derived from MRI), reliability was evaluated using the intraclass correlation coefficient (ICC). For categorical assessment, interobserver agreement for Kellgren–Lawrence (KL) grading on X-ray was evaluated using weighted Cohen’s kappa (κ), accounting for the ordinal nature of the grading system.

While cross-sectional area (CSA), volumetric MRI, and DEXA-based methods are widely regarded as reference standards for body composition assessment, their application is often limited by variability in image acquisition protocols, processing techniques, and the absence of standardized cut-off thresholds for defining skeletal muscle size. In this context, standardized single-slice measurements at defined anatomical landmarks have been shown to provide valid surrogate indicators of soft tissue composition. Accordingly, the present approach was adopted as a pragmatic and feasible method, particularly suited to retrospective imaging datasets where comprehensive volumetric assessment is not available.

### 2.1 Data analysis and used tests

Patient demographic data, including age, sex, and body mass index (BMI), were recorded. BMI was calculated as weight (kg) divided by height squared (m^2^). Imaging variables included K–L grade, MRI findings of soft tissue and cartilage degenerative changes, muscle thickness, fat thickness, and muscle-to-fat ratios. MRI findings were coded as binary outcomes (0 for “Absent,” 1 for “Present”). X-ray grades were initially treated both as categorical and later converted to binary format (0 for (grade 0) and (grade 1), and 1 for (grade 2 or higher”) to facilitate comparison with MRI.

SPSS (Statistical Package for the Social Sciences) was used for data analysis. Descriptive statistics were calculated for continuous variables to observe the distribution. Correlation analyses were conducted to examine the relationship between soft tissue thickness and knee OA severity. Pearson’s correlation analysis was performed to examine the relationships between muscle thickness, fat thickness, and degenerative changes in soft tissues and cartilage, both overall and stratified by gender. The correlation co-efficient between 0 and 0.399 was interpreted as weak, 0.4–0.599 as moderate, and 0.6–1 as strong.

A Kruskal-Wallis test was conducted to assess differences in muscle-to-fat ratio across BMI categories in all participants, as well as in gender-stratified analyses. The grade of OA was correlated with the degree of muscle and fat thickness.

Moreover, Cohen’s Kappa coefficient was calculated to assess the agreement between binary outcomes of X-ray and MRI, adjusted for chance agreement. Sensitivity, specificity, and accuracy was determined for X-ray compared to MRI, providing metrics on the diagnostic capability of X-ray imaging against the MRI gold standard. McNemar’s Test was employed as well to detect any systematic differences in the binary classifications between both modalities. In addition, Pearson’s Correlation Coefficient was used to analyze the relationship between muscle to fat ratio and degenerative changes as detected by MRI, overall and stratified by gender.

## 3 Results

### 3.1 Patient demographics

A total of 232 patients underwent both Knee X-ray and MRI (54.7%, *n* = 127) were females, and (45.3%, *n* = 105) were males. Most of them aged between 30 and 44 years (34.5%, *n* = 80) and classified in the obese BMI category (44.8%, *n* = 104). Imaging included left knee (53.9%, *n* = 125) and right knee (46.1%, *n* = 107).

### 3.2 Muscle and fat thickness

Table 1 presents data on muscle and fat thickness, and the muscle-to-fat ratio across different BMI categories stratified by gender. Muscle thickness remains relatively stable across BMI categories, with males generally having higher values than females. Additionally, Fat thickness increases with higher BMI categories, with males consistently having lower values than females. The data shows an inverse relationship between muscle-to-fat ratio and BMI category, with males showing a considerably higher ratio in the Normal category compared to females.

**TABLE 1:** Mean muscle thickness, fat thickness, and muscle-to-fat ratio across BMI categories stratified by gender.

BMI category	Muscle thickness		Fat thickness		Muscle-to-fat-ratio	
	Female	Male	Female	Male	Female	Male
Normal	5.48	5.93	3.87	2.4	1.52	3.42
Overweight	5.36	5.88	4.91	3.56	1.14	1.85
Obesity	5.51	6.02	5.92	3.98	0.997	1.81

Normality test indicated that the muscle to fat ratios are not normally distributed in the three BMI categories hence non-parametric tests were conducted.

The results of the Kruskal-Wallis test indicate a significant difference, χ^2^(2) = 29.515, *p* < 0.001, concluding that there is a difference in the muscle to fat ratio level between BMI categories. Moreover, the same test was conducted among female and male participants separately. The analysis indicates a statistically significant difference in the level of Muscle to fat ratio between the different BMI categories among females [χ^2^(2) = 40.405, *p* < 0.001] and males [χ^2^(2) = 17.954, *p* < 0.001].

### 3.3 Interobserver agreement

Interobserver agreement demonstrated good reliability for the quantitative MRI measurements. The intraclass correlation coefficient (ICC) for muscle thickness was 0.82 (95% CI: 0.75–0.88), and for fat thickness was 0.87 (95% CI: 0.81–0.92), indicating consistent measurements between observers. Interobserver agreement demonstrated good reliability for the quantitative measurements. For X-ray assessment, interobserver agreement for KL grading demonstrated good to excellent agreement (κ = 0.84), reflecting reliable classification of osteoarthritis severity between observers.

### 3.4 Degenerative changes

Pearson’s correlation analysis demonstrated a weak negative correlation between the muscle to fat ratio and soft tissue degenerative changes [*r*(232) = −0.129, *p* = 0.049] and cartilage degenerative changes [*r*(232) = −0.170, *p* = 0.009] for all participants.

**FIGURE 2:**
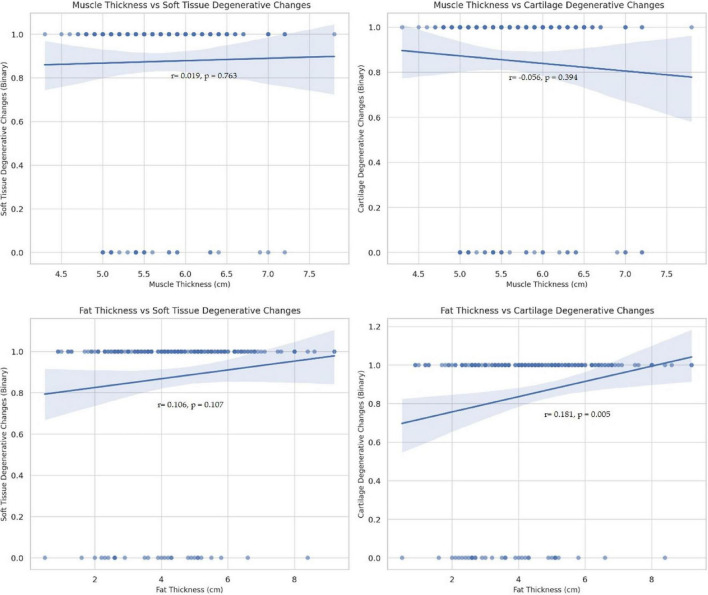
Correlation of muscle, fat thickness, and degenerative changes (cartilage and soft tissue).

The correlations between muscle thickness, fat thickness, muscle-to-fat ratio, and degenerative changes are summarized in Table 2 and illustrated in Figure 2. A weak positive correlation was found between muscle thickness and soft tissue degenerative changes, which was not statistically significant [*r*(232) = 0.019, *p* = 0.763]. Similarly, the weak negative correlation between muscle thickness and cartilage degenerative changes was also not statistically significant [*r*(232) = −0.056, *p* = 0.394]. While the fat thickness and soft tissue degenerative changes had a weak positive correlation, which did not reach statistical significance [*r*(232) = 0.106, *p* = 0.107]. However, a weak positive correlation between fat thickness and cartilage degenerative changes was statistically significant [*r*(232) = 0.181, *p* = 0.005].

**TABLE 2:** Correlation analysis of muscle thickness, fat thickness, and muscle-to-fat ratio with soft tissue and cartilage changes according to gender.

Participant group	Muscle thickness		Fat thickness		Muscle-to-Fat Ratio	Muscle-to-Fat Ratio
	Soft tissue changes	Cartilage changes	Soft tissue changes	Cartilage changes	Soft tissue changes	Cartilage changes
All participants	Weak positive correlation	Weak negative correlation	Weak positive correlation	Weak positive correlation	Weak negative correlation soft tissue changes *p* = 0.049	Weak negative correlation cartilage changes *p* = 0.009
*P*-value	Not statistically significant = 0.763	Not statistically significant = 0.394	Not statistically significant = 0.107	Statistically significant = 0.005		
Male	Weak negative correlation	Weak negative correlation	Weak positive correlation	Weak positive correlation	Weak negative correlation soft tissue changes *p* = 0.085	Weak negative correlation cartilage changes p = 0.065
*P*-value	Not statistically significant = 0.864	Not statistically significant = 0.319	Not statistically significant = 0.945	Not statistically significant *p* = 0.167		
Female	Weak positive correlation	Very weak positive correlation	Moderate positive correlation	Moderate positive correlation	Weak negative correlations soft tissue changes *p* < 0.001	Weak negative correlations cartilage changes *p* = 0.065
*P*-value	Not statistically significant = 0.922	Not statistically significant = 0.922	Statistically significant = 0.005	Statistically significant = 0.007		

Similarly for male patients weak negative correlation between the muscle to fat ratio vs. soft tissue degenerative changes [*r*(105) = −0.169, *p* = 0.085] and cartilage degenerative changes [*r*(105) = −0.181, *p* = 0.065]. On the other hand, for female patients’ weak negative correlations were observed between their muscle to fat vs. soft tissue degenerative changes [*r*(127) = −0.306, *p* < 0.001] and cartilage degenerative changes [*r*(105) = −0.181, *p* = 0.065].

**FIGURE 3:**
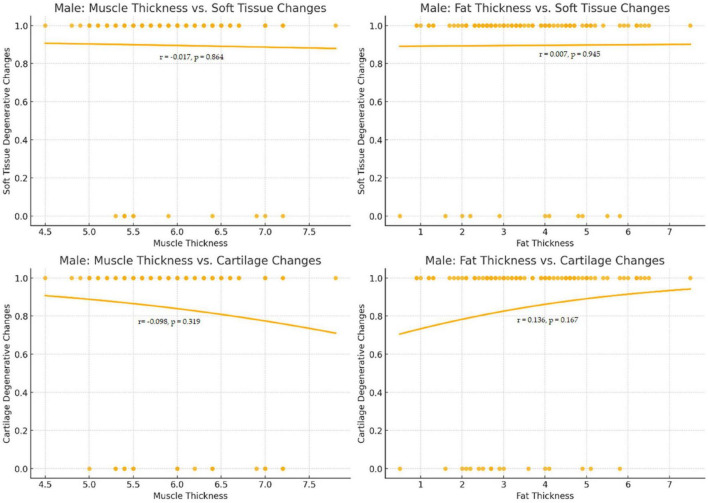
Male correlation of muscle, fat thickness, and degenerative changes (cartilage and soft tissue).

Pearson’s correlation analysis was used to evaluate the relationships between muscle thickness, fat thickness, and degenerative changes in soft tissues and cartilage among male and female participants separately (see Figure 3) the results indicated:

For males:

- A weak negative correlation between muscle thickness and soft tissue changes, not statistically significant [*r*(232) = −0.017, *p* = 0.864]. A weak negative correlation between muscle thickness and cartilage changes, not statistically significant [*r*(232) = −0.098, *p* = 0.319].

- A weak positive correlation between fat thickness and soft tissue changes, also not statistically significant [*r*(232) = 0.007, *p* = 0.945]. A weak positive correlation between fat thickness and cartilage changes, not statistically significant [*r*(232) = 0.136, *p* = 0.167].

**FIGURE 4:**
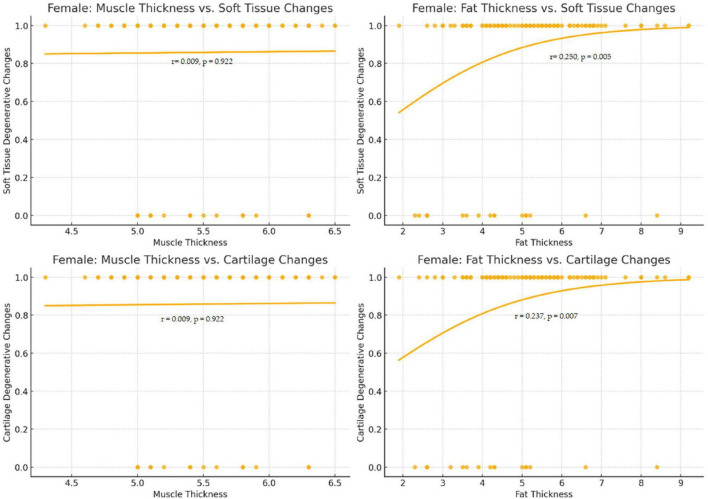
Female correlation of muscle, fat thickness, and degenerative changes (cartilage and soft tissue).

For females, as in Figure 4.

- A weak positive correlation between muscle thickness and soft tissue changes, not statistically significant [*r*(232) = 0.009, *p* = 0.922]. A weak positive correlation between muscle thickness and cartilage changes, not statistically significant [*r*(232) = 0.009, *p* = 0.922].

- A weak positive correlation between fat thickness and soft tissue changes, statistically significant [*r*(232) = 0.250, *p* = 0.005]. A weak positive correlation between fat thickness and cartilage changes, statistically significant [*r*(232) = 0.237, *p* = 0.007].

### 3.5 Appropriateness of X-ray criteria

The relation between X-ray grading and MRI findings for soft tissue and cartilage degenerative changes was significant. For soft tissue degenerative changes, the Chi-squared analysis yielded significant results, X^2^ (1, *N* = 232) = 31.85, *p* < 0.001), with a Cramér’s V of 0.371 indicating a moderate strength of association. Similarly, for cartilage degenerative changes, the test results were X^2^ (1, *N* = 232) = 41.55, *p* < 0.001), with a Cramér’s V of 0.423, also suggesting a moderate strength of association. These findings indicate that changes in X-ray grades are moderately associated with the degenerative changes observed in MRI scans.

The Chi-square analysis between the X-ray grading and combined MRI findings yielded significant results, X^2^ (1, *N* = 232) = 36.67, *p* < 0.001), with a Cramér’s V of 0.398, suggesting a moderate strength of association.

**FIGURE 5:**
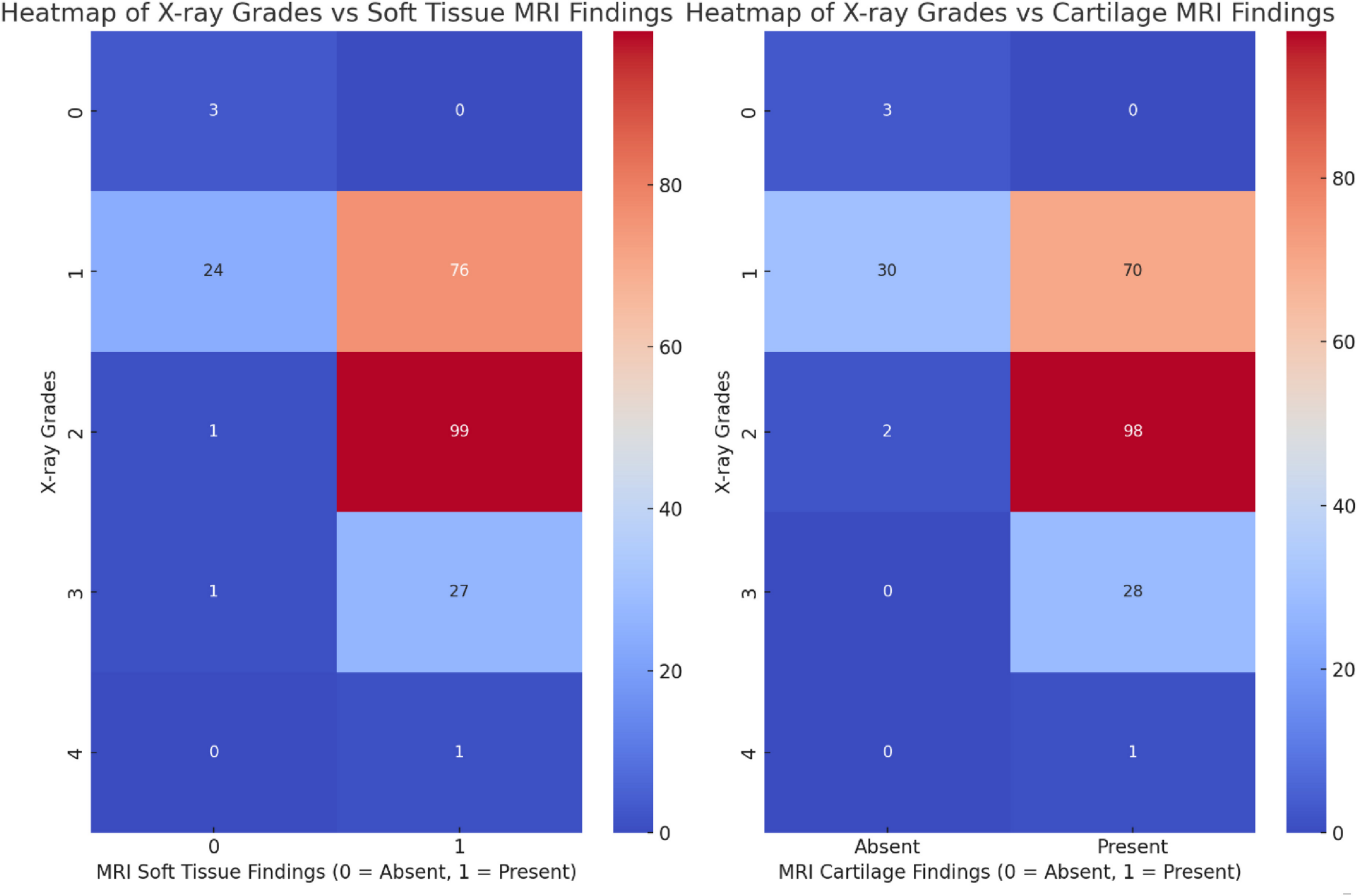
The relationship between X-ray grades and MRI findings for both soft tissue and cartilage degenerative changes.

The heatmaps in Figure 5, visually represent the relationship between X-ray grades and MRI findings for both soft tissue and cartilage degenerative changes. The numbers within each cell indicate the count of observations corresponding to each combination of X-ray grade and MRI finding status.

- Column 0: represent discord between two modalities, where x-ray indicates positive finding and MRI not.

- Column 1: shows concord when both X-ray and MRI indicates findings.

Cohen’s Kappa coefficient analysis was performed to evaluate the level of agreement between binary classifications derived from X-ray and MRI diagnostic methods. The Cohen’s Kappa showed there was fair agreement between X-ray and MRI soft tissue changes with *k* = 0.266, *p* < 0.001 and also fair agreement between the X-ray and MRI degenerative changes with *k* = 0.327, *p* < 0.001. Further, a fair level of agreement *k* = 0.273, *p* < 0.001, was revealed between X-ray and combined MRI findings.

**FIGURE 6:**
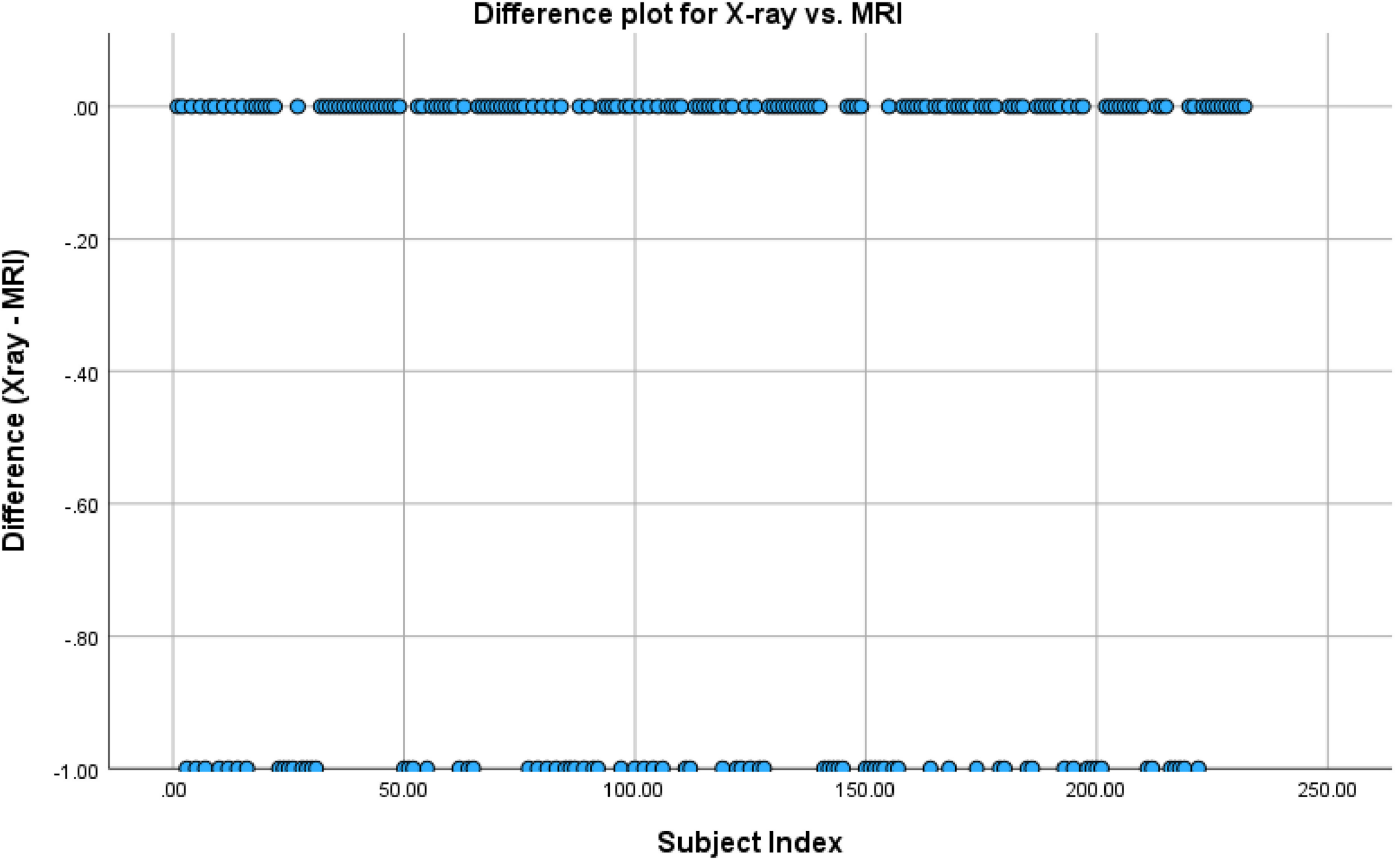
Difference plot for X-ray compared to MRI to evaluate the consistency of diagnostic outcomes.

The McNemar’s test was conducted to evaluate the consistency of diagnostic outcomes between X-ray and combined MRI. The results indicated a statistically significant difference (p < 0.001) in detection capabilities between the two diagnostic methods (see Figure 6).

In a comparative analysis between X-ray and MRI, the diagnostic performance of X-ray was evaluated in terms of sensitivity, specificity, accuracy, and agreement. The sensitivity of the X-ray was approximately 62.6% and a specificity of 100%. Yielding an overall accuracy of 66.8%.X-ray produced no false-positives but the moderate sensitivity reflects a high false-negative rate.

**FIGURE 7:**
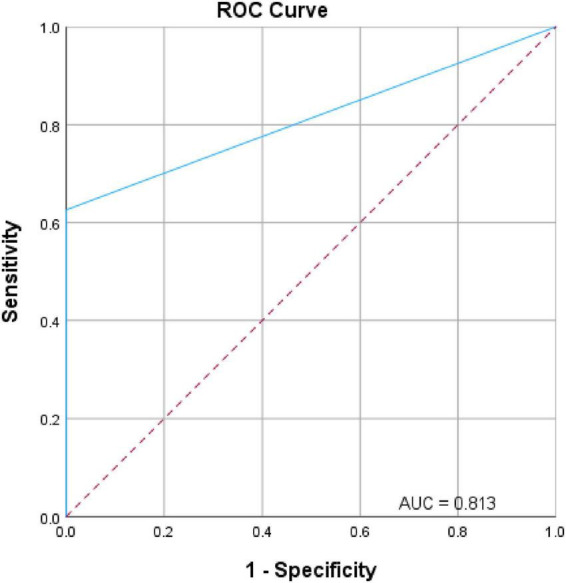
Receiver operating characteristic (ROC) curve, illustrating the diagnostic accuracy of the X-ray compared to MRI.

Figure 7 shows the Receiver Operating Characteristic (ROC) curve, which illustrates the diagnostic accuracy of the X-ray compared to MRI findings. The ROC curve plots the True Positive Rate (Sensitivity) against the False Positive Rate (1 − Specificity) for different possible thresholds of the X-ray grades.

The area under the curve (AUC) is approximately 0.813, indicating very good diagnostic ability of the X-ray to discriminate between the presence and absence of conditions as determined by MRI. An AUC of 1.0 would indicate perfect diagnostic accuracy, while an AUC closer to 0.5 suggests no diagnostic ability beyond random chance.

## 4 Discussion

### 4.1 Principal findings and study context

This study examined the relationship between MRI-assessed muscle and fat characteristics and knee OA severity, in relation to radiographic findings, with attention to gender differences. By integrating X-ray and MRI assessment, the study highlights the influence of BMI and muscle–fat balance on knee degenerative changes, particularly among female patients.

### 4.2 Muscle–fat–bone interaction in Knee OA

Previous studies discussed the interconnected role of bones, muscles, and adipose tissue in musculoskeletal health. Spanoudaki et al highlighted that OA, sarcopenia and obesity should be considered and managed as a unified condition rather than isolated entities (14). Our findings align with this concept, supporting the relevance of muscle–fat balance in knee OA and reinforcing the importance of evaluating periarticular soft tissues alongside structural changes.

The association between muscle strength and knee OA symptoms, disease onset and progression has been widely reported (15). Moreover, the role of neuromuscular function in the OA disease cycle has also been clarified (16). According to Rice DA et al., quadriceps weakness is clinically significant in people with OA, being linked to physical disability and found to be a risk factor for the development and advancement of joint degeneration (17). The findings of the present study indicate obese females and males have significantly lower muscle to fat ratio compared to normal weight.

### 4.3 Obesity, fat thickness, and gender differences

Obesity has consistently been associated with an increased risk of Knee OA, evidence suggesting that fat thickness plays a more prominent role than lean thickness, particularly among women (18). Studies in body compositions assessment, using Dual-energy X-ray absorptiometry (DEXA), demonstrated reduced muscle thickness and increased fat thickness in individuals with knee OA. DEXA analysis demonstrated that individuals with knee OA exhibited reduced muscle thickness and increased fat thickness, providing insight into the influence of body composition on knee degenerative changes variables affect knee degenerative change. They found that the DEXA measurement of the knee osteoarthritic group’s body composition indicates a significant loss in overall muscle thickness as compared to the control group (19). In the present study, the stronger associations observed in female participants support the hypothesis that adiposity-related metabolic and inflammatory mechanisms may contribute more substantially to knee degeneration in women, whereas biomechanical factors may be more relevant in men. These findings reinforce the need for gender-specific approaches to the prevention and management of knee OA.

The risk of knee OA has consistently been associated with obesity. Nur et al. demonstrated in their study of postmenopausal women, that body weight has a significant positive correlation with knee OA. Among the components of body weight, fat thickness showed a stronger and more significant association with knee OA than lean thickness. This finding indicates that the link between obesity and KOA is primarily driven by fat thickness, highlighting the critical role of the systemic metabolic effects of adiposity in this relationship (20). In the same context, Solanki et al found that each 5 kilograms increase in body weight resulted with a 34% increase in the probability of knee OA and total knee replacement (21). These observations are consistent with the present study, in which adiposity-related measures showed significant associations with knee degenerative changes, particularly among female participants.

The findings suggest that muscle thickness is relatively stable across BMI categories, whereas the muscle-to-fat ratio is more strongly influenced by increasing fat thickness. This observation may reflect the influence of factors such as hormonal differences, metabolic profile, and physical activity patterns, which may differentially affect body composition across genders. This observation suggests that muscle thickness is less influenced by BMI and more likely related to other factors such as genetic predisposition, hormonal differences, or physical activity levels.

Conversely, the fat thickness increases with higher BMI categories for both women and men. Similarly, Karlsson et al., in their study found all genders with idiopathic knee OA have a phenotype with higher BMD, higher BMI, relatively lower lean body thickness, and relatively higher fat thickness. Additionally, men tend to have a larger skeletal structure. While the increased BMD could enhance the stability of prosthesis fixation, the higher BMI may result in a higher joint load (22).

The findings reinforce the importance of considering both muscle and fat components when assessing the implications of BMI on health. The decreasing muscle-to-fat ratio with higher BMI categories, especially in females, highlights the need for targeted interventions aimed at reducing fat thickness while preserving or increasing muscle thickness to improve overall health outcomes.

The findings suggest that knee degenerative changes are highly affected by muscle-to-fat ratio and fat thickness, which appear more pronounced in females, implying that the female body composition has a higher effect on degenerative changes than males.

In the present study, females exhibited stronger associations between adiposity-related measures and knee degenerative changes compared with males. This observation is consistent with previous work by Visser AW et al., who reported that fat thickness demonstrated a stronger association with knee OA in women, whereas skeletal muscle thickness showed a greater association in men (23).

### 4.4 Imaging interpretation: X-ray versus MRI

Although radiography remains the standard imaging modality for knee OA assessment due to its accessibility and low cost, its limited ability to characterize soft tissue and early cartilage changes is well recognized. MRI provides complementary information by enabling detailed evaluation of cartilage and periarticular soft tissues that may not be adequately captured on radiographs alone. MRI is thought to be a more accurate method of tracking the progression of the degenerative changes. According to a study assessing the correlation between cartilage loss on MRI and joint narrowing on X-rays, radiography is not a sensitive measure and would likely miss a significant percentage of knees with cartilage loss if used alone for knee OA (24). MRI is a valuable diagnostic technique. In a recent systematic analysis, MRI revealed a sensitivity of 0–86% for identifying early grades and up to 98% for advanced grades, and a specificity of 48–95% for early grades and up to 100% for advanced grades (25). The current study showed the high specificity of radiography and indicates a zero false-positive rate. The current study showed sensitivity of 62.6% and accuracy of 66.8% reflecting a need for MRI to support accurate diagnosis. Study done by Stephen et al. supported the same result, suggested that more advanced imaging techniques like MRI are likely necessary to inform treatment decisions (26), as regular radiography has low sensitivity and is better to detect severe arthritis (26, 27).

### 4.5 Clinical implications and future directions

These findings highlight the importance of considering both muscle and fat tissue components when interpreting the relationship between BMI on knee OA, particularly among female patients. However, as this study is based on imaging-derived measures without corresponding clinical outcomes, the results should be interpreted as radiological associations rather than indicators of symptom severity or functional impairment. Nevertheless, the findings suggest that body composition may play a role in structural joint changes, warranting further investigation incorporating clinical endpoints. The complementary use of MRI alongside radiography may enhance the characterization of joint pathology and improve diagnostic confidence, thereby supporting more informed clinical assessment within an imaging context.

## 5 Limitations

This study has limitations that should be considered when interpreting the findings. The sample size was relatively small, which may limit the generalizability of the results. The retrospective design further constrained the availability of relevant clinical data, including patient-reported outcomes such as pain severity and functional status; therefore, the findings should be interpreted as imaging-based associations rather than direct indicators of clinical impact.

In addition, periarticular soft tissue composition was assessed using indirect measurements of muscle and fat thickness rather than volumetric techniques or established reference methods such as dual-energy X-ray absorptiometry (DEXA), which may introduce measurement limitations. Although consistent anatomical landmarks were used to improve reproducibility, the absence of standardized segmentation approaches may affect precision.

Furthermore, MRI was used as the reference imaging modality in the absence of arthroscopic or histopathological validation, which may introduce verification bias, particularly in the assessment of diagnostic performance.

## 6 Conclusion

Decreasing muscle-to-fat ratio with higher BMI categories, especially in females, highlights the need for targeted interventions aimed at reducing fat thickness while preserving or increasing muscle thickness to improve overall health outcomes. For future research, it is recommended to do prospective studies using x-ray, MRI and DEXA scans with consideration of increasing the sample size to enhance reliability and generalizability. Moreover, establishing a correlation between research outcomes and arthroscopy as a more accurate gold standard would validate the results.

## Data Availability

The raw data supporting the conclusions of this article are available from the corresponding author, subject to institutional and ethical restrictions.
