# Correction: Association of MRI-derived muscle-fat composition with knee osteoarthritis severity: a gender-stratified X-ray and MRI correlation study

**DOI:** 10.3389/fmed.2026.1924209

**Published:** 2026-07-27

**Authors:** Mohammad Al-Harbi, Anas Hamdoun, Zuhal Y. Hamd, Shaden Alhegail, Ammar Mallisho, Razan Salih, Mohamed Abuzaid, Wiam Elshami

**Affiliations:** 1 Internal Medicine Department, College of Medicine, Princess Nourah bint Abdulrahman University, Riyadh, Saudi Arabia; 2 Department of Medical Imaging, King Abdullah Bin Abdul-Aziz University Hospital, Riyadh, Saudi Arabia; 3 Department of Radiological Sciences, College of Health and Rehabilitation Sciences, Princess Nourah bint Abdulrahman University, Riyadh, Saudi Arabia; 4 Department of Medical Diagnostic Imaging, College of Health Sciences, University of Sharjah, Sharjah, United Arab Emirates; 5 Research Institute for Medical and Health Sciences, University of Sharjah, Sharjah, United Arab Emirates

**Keywords:** knee osteoarthritis, magnetic resonance imaging, radiography, Kellgren-Lawrence grading, muscle-to-fat ratio, body composition, cartilage degeneration, soft tissue changes

An incorrect Funding statement was provided. The statement previously declared:

“The author(s) declared that financial support was not received for this work and/or its publication.”

The updated funding statement appears below:

## Funding

The author(s) declared that financial support was received for this work and/or its publication. This research was funded by Princess Nourah bint Abdulrahman University Researchers Supporting Project number (PNURSP2026R850), Princess Nourah bint Abdulrahman University, Riyadh, Saudi Arabia.

The original version of this article has been updated.

